# Pbx homeodomain proteins pattern both the zebrafish retina and tectum

**DOI:** 10.1186/1471-213X-7-85

**Published:** 2007-07-16

**Authors:** Curtis R French, Timothy Erickson, Davon Callander, Karyn M Berry, Ron Koss, Daniel W Hagey, Jennifer Stout, Katrin Wuennenberg-Stapleton, John Ngai, Cecilia B Moens, Andrew J Waskiewicz

**Affiliations:** 1 Department of Biological Sciences, University of Alberta, Edmonton, T6G2E9, Canada; 2 Division of Basic Sciences, Fred Hutchinson Cancer Research Center, Seattle, Washington, 98115, USA; 3 Department of Molecular and Cellular Biology, Functional Genomics Laboratory, and Helen Wills Neuroscience Institute, University of California, Berkeley, California, 94720, USA; 4 Affymetrix Inc., Santa Clara, California, 95051, USA

## Abstract

**Background:**

*Pbx *genes encode TALE class homeodomain transcription factors that pattern the developing neural tube, pancreas, and blood. Within the hindbrain, Pbx cooperates with Hox proteins to regulate rhombomere segment identity. Pbx cooperates with Eng to regulate midbrain-hindbrain boundary maintenance, and with MyoD to control fast muscle cell differentiation. Although previous results have demonstrated that Pbx is required for proper eye size, functions in regulating retinal cell identity and patterning have not yet been examined.

**Results:**

Analysis of retinal ganglion cell axon pathfinding and outgrowth in *pbx2/4 *null embryos demonstrated a key role for *pbx *genes in regulating neural cell behavior. To identify Pbx-dependent genes involved in regulating retino-tectal pathfinding, we conducted a microarray screen for Pbx-dependent transcripts in zebrafish, and detected genes that are specifically expressed in the eye and tectum. A subset of Pbx-dependent retinal transcripts delineate specific domains in the dorso-temporal lobe of the developing retina. Furthermore, we determined that some Pbx-dependent transcripts also require Meis1 and Gdf6a function. Since *gdf6a *expression is also dependent on Pbx, we propose a model in which Pbx proteins regulate expression of the growth factor *gdf6a*, which in turn regulates patterning of the dorso-temporal lobe of the retina. This, in concert with aberrant tectal patterning in *pbx2/4 *null embryos, may lead to the observed defects in RGC outgrowth.

**Conclusion:**

These data define a novel role for Pbx in patterning the vertebrate retina and tectum in a manner required for proper retinal ganglion cell axon outgrowth.

## Background

Vertebrate embryos use a combination of transcription factors to specify position along the anterior-posterior (A-P) axis. Of particular importance is the Pbx (pre-B cell leukemia homeobox)-family of TALE (Three Amino acid Loop Extension)-class transcription factors, which are required globally to pattern the A-P axis of the developing vertebrate embryo. Using mouse knockout and zebrafish knockdown models, researchers have shown that Pbx proteins are required to specify cell fate in the midbrain, hindbrain, somites, pancreas, and blood [1-4]. In the hindbrain, trimeric DNA-binding complexes of Pbx, Hox and Meinox (Meis/Pknox) proteins specify rhombomere identity. In the midbrain, Pbx cooperates biochemically with Engrailed (Eng) proteins to maintain both midbrain-hindbrain and the diencephalic-mesencephalic boundaries [2,5].

Pbx clearly also plays a role in patterning regions outside of the midbrain and hindbrain. For example, mouse knockouts have demonstrated a critical role for Pbx during pancreatic development, in which interactions between Pbx and Insulin promoter factor 1 (Ipf1) are required for transcriptional activity, and subsequent expansion of pancreatic cell lineages [3]. Pbx also plays a role in the development of blood, as Pbx – Prep1 (also known as Pknox1) complexes are required for the production of normal populations of CD4 and CD8 T-lymphocytes [6]. Furthermore, Pbx – Meis complexes have been implicated in megakaryocyte differentiation in rats, through the ability to initiate transcription from the platelet factor 4 (PF4) promoter [4]. Recently, it has been shown during the development of skeletal muscle that Pbx is constitutively bound to the *Myogenin *promoter, can bind directly to the bHLH transcription factor MyoD, and is thus required for the development of muscle cell fates [7]. A zebrafish mutant, *lazarus (lzr)*, that contains a null mutation in the *pbx4 *gene [8], displays global defects in embryonic patterning including hindbrain, muscle, blood, and midbrain tissues.

The Meinox (Meis/Pknox/Prep) family of TALE-class transcription factors are well characterized as DNA-binding cofactors for both Pbx and Hox proteins [9]. Zebrafish contain at least six *meis *genes that are expressed in dynamic and tissue specific manners [1,10-12]. In addition, zebrafish possess at least two *pknox/prep *genes that are both widely expressed [13]. *meis *genes are prominently expressed in the developing retina [1,10-12] suggesting a role for *meis *in regulating eye formation or patterning. In zebrafish, overexpression of *hoxb2 *throughout the embryo causes ectopic expression of hindbrain markers exclusively within the retina, demonstrating the existence of retinal specific Hoxb2 competency factors [14]. Pbx and Meinox proteins function as two of these retinal Hoxb2-competency factors, demonstrating that both Pbx and Meis can function in the retina [1,8,15,16]. The idea that Meinox proteins play a role in eye formation is supported by studies in Drosophila, where the *pbx *homologue *extradenticle *(*exd*) and the *meis *homologue *homothorax *(*hth*) inhibit eye formation [17], and in mice where the *Pknox1 *hypomorphic and *Meis1 *knockout phenotypes include defects in retinal development. [16,18]

Zebrafish make an excellent model for the study of retinal development. The optic primordium is distinct from surrounding tissues as early as 12 hours post fertilization (hpf), and by 24 hpf, eyecups have developed to include lens tissue and the surrounding neural retina [19]. By 30 hpf, the first post-mitotic neurons have differentiated [20] and by 50 hpf, lamination is evident across the retina. Two partially redundant *pbx *genes, *pbx2 *and *pbx4*, are expressed during early development when the optic primordium and optic cup are developing. The other zebrafish *pbx *genes, *pbx1 *and *pbx3.1*, are expressed more strongly starting at 24 hpf. Thus, inhibition of Pbx2 and Pbx4 in a homozygotic *lazarus *(*lzr*) (*pbx4 *mutant) embryo allows for analyses of development in the absence of Pbx function, prior to 24 hpf (see methods). The retina of *lazarus (pbx4*^-/-^*) *zebrafish embryos is noticeably smaller than wild type embryos, but no analysis of patterning has yet been undertaken. This manuscript provides an analysis of Pbx and Meis function in the developing zebrafish retina, as well as a role for Pbx in patterning the tectum, and includes a microarray screen for *pbx *dependent transcripts in zebrafish to identify gene regulatory networks downstream of this critical homeodomain protein. We propose a model in which Pbx functions to specify regional identity in the developing zebrafish retina via activation of the dorsal/temporal specific growth factor, *gdf6a*.

## Results

### Retinal laminar structure is normal but retinal ganglion cell axon outgrowth is aberrant in *pbx2/4 *null embryos

**Figure 1:**
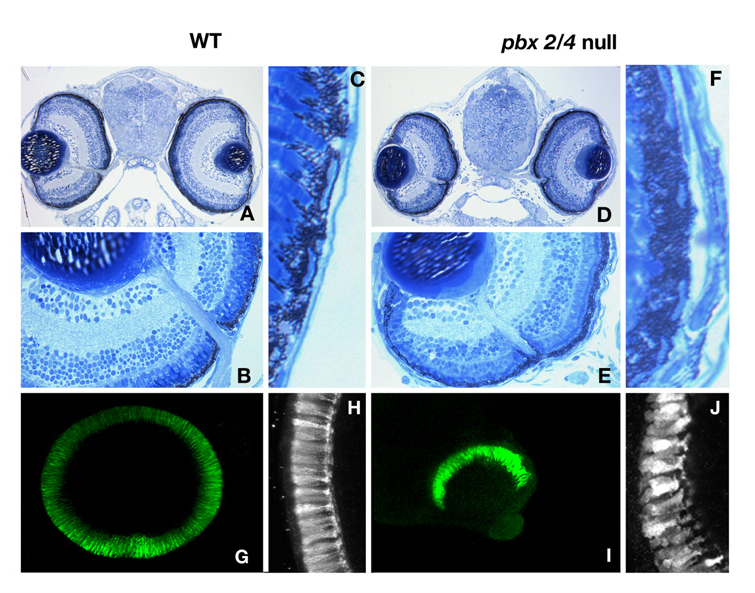
The laminar structure of *pbx2/4 *null eyes is normal after 5 days of development. All laminar layers are present and in both wild type (A and B) and, and *lzr *mutants injected with *pbx2/4 *morpholinos (D and E). We note a consistent decrease in eye size in *pbx2/4 *null embryos, but the proportion of eye area occupied by the retinal pigmented epithelium in morphants is significantly increased (F verses C). The relative position of the optic nerve is similar in both wild type and morphant embryos. The retinal photoreceptor layer is absent in the ventral domain of the eye in *lzr *mutants injected with *pbx2/4 *morpholinos (I) when compared to wildtype (G) and is disorganized (compare H, wildtype, with J, *lzr *mutants injected with *pbx2/4 *morpholinos). Photoreceptor labeling was accomplished using the zpr-1 monoclonal antibody.

Previous studies have demonstrated a clear role for the homeodomain transcription factor Pbx in regulating cell fates in the hindbrain and at the midbrain-hindbrain boundary. The eyes of *lazarus *(*pbx4*^-/-^) embryos are distinctly smaller than their wild type counterparts, indicating that Pbx is required in this tissue as well [8]. As a first step towards dissecting the role of Pbx proteins in eye development, we compared eye morphology between wild type and *pbx2/4 *null (*pbx4*^-/-^; *pbx2/4 *MO) at 5 days post-fertilization (dpf). The laminar structure of the retina appears relatively normal in *pbx2/4 *null embryos (compare wild types in Figure 1A, B to *pbx2/4 *nulls in Figure 1D, E) and the optic nerve exits the eye in the correct location. All nuclear layers are clearly visible in *pbx2/4 *null eyes, including the inner most retinal ganglion cell layer, and the outermost nuclear layer containing the photoreceptor cells. The retinal pigmented epithelium (RPE) of *pbx2/4 *null embryos is thickened (9.14 +/- 1.9 % of total eye area, Figure 1F) in comparison to wild type controls (5.95 +/- 0.3 % of total eye area, p < 0.05, Figure 1C), indicating that Pbx has a role in regulating RPE differentiation. There is also a distinct change in layer morphology, at the interface of the RPE and photoreceptor layer. In addition to thickened RPE, *pbx2/4 *null embryos have a disorganized photoreceptor layer (compare Figure 1H with 1J), which likely contributes to the aberrant morphology seen at the interface. These data suggest that while *pbx2/4 *null eyes are smaller than their wild type counterparts, Pbx proteins are not required for establishing the laminar structure of the eye.

**Figure 2:**
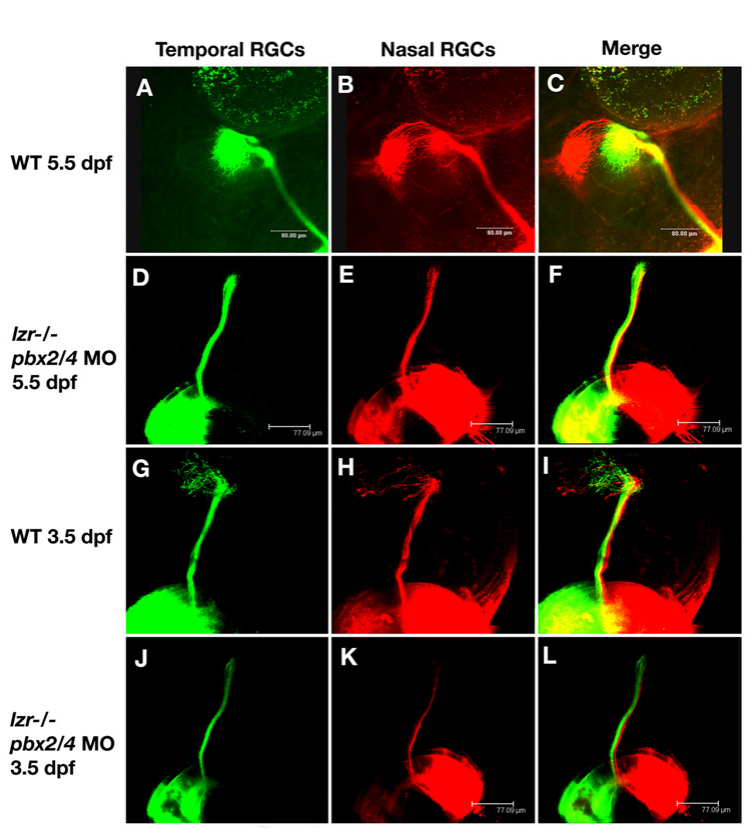
*pbx2/4 *null embryos exhibit RGC axonal outgrowth defects. In wild type embryos at 5.5 dpf, temporal RGC axons map to the anterior optic tectum (A) and (C), while nasal RGC axons map to the posterior region of the optic tectum (B) and (C). This is also seen at 3.5 dpf (G-I) although the connections on the tectum are more diffuse. In *lzr*-/- mutants injected with *pbx2/4 *morpholinos, both posterior and anterior RGC axons fail to map onto the optic tectum at both 3.5 dpf (J-L) and 5.5 dpf (D-F).

In wild type embryos, retinal ganglion cells innervate the optic tectum to form an inverse topographic map of the eye. Axons that originate in the temporal retina innervate the anterior tectum (Figure 2A, C), while axons that originate in the nasal retina innervate the posterior tectum (Figure 2B, C). In embryos lacking both *pbx2 *and *pbx4*, no topographic mapping of the RGC axons is observed at 3.5 or 5.5 dpf. In the majority of embryos (6/7 at 3.5dpf, 11/14 5.5dpf), the primary axon bundle projects toward the optic tectum, but axons appear stalled before entry to the tectum as no branching or outgrowth is observed (Figure 2D–F, 2J–L). These results suggest that Pbx proteins regulate transcriptional pathways that control RGC axonal outgrowth and topographic mapping.

### Whole embryo microarray analysis of Pbx mutants identifies known hindbrain patterning genes

In the presumptive hindbrain, Pbx2 and Pbx4 functions are critically required for initiation of *early growth regulator 2b *(*egr2b*, previously *krox20*), *musculoaponeurotic fibrosarcoma oncogene family protein B (mafb*, previously *valentino*), *homeobox b1A (hoxb1a)*, *hoxa2b*, and *hoxb2*. To identify Pbx-dependent transcripts that might regulate retinotectal patterning, we conducted microarray comparisons between 18 hpf control embryos (*pbx2 *MO injected) and *pbx2/4 *null embryos (mz *lzr*; *pbx2 *MO). Each array contained 8448 cDNA probes, including 768 controls spots (e.g. negative, positive, and normalization controls) derived from a normalized mixed stage zebrafish cDNA library [21]. We identified 31 transcripts that were downregulated by at least 2 fold and 284 transcripts that were downregulated by at least 1.5 fold. We repeated these analyses using two independent biological replicates at 18 hpf, two biological replicates at 28 hpf, and using two biological replicates of 18 hpf zygotic *lzr *embryos injected with *hoxb1a *and *hoxb1b *morpholinos, as these phenotypically mimic the *pbx2/4 *null phenotype in the hindbrain [2]. Overall, we identified 366 ESTs that were downregulated by 1.5 fold in at least two datasets. Student T-tests were conducted on genes of interest based on annotation and known expression patterns. Focusing on clones that were affected at 18 hours, we generated antisense probes and sequence information for 132 EST clones. We examined 70 of these clones by *in situ *hybridization, to which 33 showed Pbx dependence at 18 hpf. Among these clones, we identified *egr2b*, *hoxb3a*, *mafb*, and *engrailed 2b *(*eng2b*, previously *eng3) *(Table 1). Each of these clones is expressed in either the midbrain-hindbrain boundary or the hindbrain at 18 hpf and were previously shown to be downregulated in *pbx2/4 *null embryos [2]. Given this validation, the microarray is clearly capable of identifying *bona fide *Pbx dependent transcripts.

**Table 1:** Microarray identified Pbx-dependent transcripts expressed in the hindbrain, eye, and tectum

gene	Expression pattern	Accession	Average fold change 18hpf	M	T
*egr2b*	rhombomere 3, 5	NM_130997	7.98	3.00	35.71
*mafb*	rhombomere 5	AB006322	2.09	1.06	16.63
*meis3*	central nervous system, neural crest	NM_131778	1.88	0.91	5.54
*eng2b*	midbrain/hindbrain boundary	NM_131040	2.94	1.33	3.17
*eng2a*	mid/hindbrain boundary	NM_131044	1.78	0.83	3.14
*aldh1a2*	posterior retina, somites	NM_131850	1.80	0.83	3.46
*tbx5*	heart, pectoral fin, posterior retina	NM_130915	1.88	0.59	14.80
*hmx3b*	lens, dorsal retina	NM_001045371	1.38	0.46	18.14
*fabp7*	eye, tectum, midbrain, hindbrain	NM_021272	2.10	1.07	16.63
*efna2*	tectum, midbrain, mid/hindbrain boundary	BC096783	1.37	0.46	2.87
*nat10*	eye, tectum, midbrain, somites	BC057462	1.39	0.48	6.53

M, log2 (WT/lzr, pbx mo)

T, ratio of mean difference/standard deviation

### Microarray and whole mount *in situ *analysis demonstrates that Pbx proteins regulate gene expression in the developing retina

**Figure 3:**
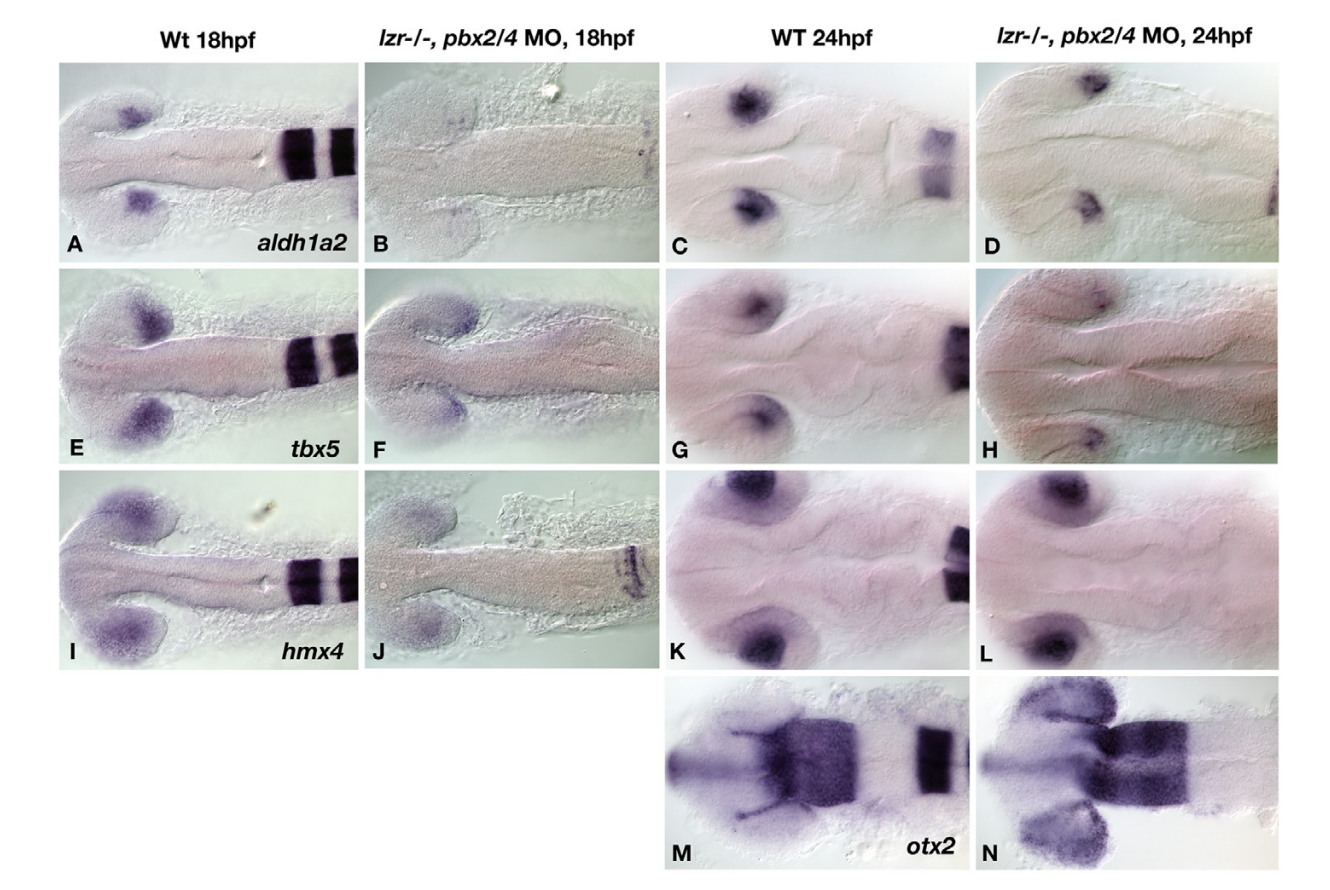
Gene expression analysis via *in situ *hybridization shows altered expression of Pbx dependent transcripts in *pbx2/4 *null embryos. The expression of *aldh1a2*, *tbx5*, and *hmx4 *is reduced in *pbx2/4 *null embryos (B, F, and J), when compared to wild type (A, E, and I) at 18 hpf. At 24 hpf, *aldh1a2 *and *tbx5 *show reduced expression in *pbx2/4 *null embryos (D and H) when compared to wildtype (C and G), while the expression of *hmx4 *is unaffected (K and L). The expression of *otx2 *is limited to the RPE at the periphery of the eye in wild type embryos at 24 hpf (M), and is expanded to the posterior retina in *pbx2/4 *null embryos (N). All embryos were hybridized with the probe indicated, as well as *egr2b*, as an indicator of the level of Pbx function.

Microarray analysis also uncovered Pbx dependent transcripts expressed specifically in regions outside of the hindbrain, notably including genes expressed in the muscle [22] and in the retina and tectum (described in this manuscript). Of the 132 ESTs studied, 13 are specifically expressed in the retina, tectum, or both. Three genes, *aldehyde dehydrogenase 1 family, member A2 (aldh1a2*, previously *raldh2*), *T-box 5 *(*tbx5*), and *hmx4 *(ortholog of chick *soho1*) are expressed in the dorsal retina. The transcripts for *aldh1a2*, and *tbx5 *are expressed in the dorsal/temporal retina at both 18 and 24 hpf (Figure 3A and 3C, E and 3G), while *hmx4 *is specific to lens and dorsal retina (Figure 3I and 3K). To examine retinal patterning in *pbx2/4 *null embryos, we performed *in situ *hybridization with these three putative Pbx dependent genes as well as *orthodenticle homolog 2 *(*otx2*), a marker of retinal pigmented epithelium and the optic tectum at 24 hpf [23,24]. Three of these genes, *aldh1a2*, *tbx5*, and *hmx4 *are strongly downregulated in *pbx2/4 *null embryos at 18 hpf (Figure 3B, F, and 3J), thus supporting the microarray findings. At 24 hpf, the expression of *aldh1a2 *and *tbx5 *is downregulated (Figure 3D and 3H), while the expression of *hmx4 *is unaffected (Figure 3L). Although we do not observe any change in the expression of *otx2 *in the tectum of *pbx2/4 *null embryos at 24 hpf, there is a marked expansion of the domain of *otx2 *expression to include the entire eye in these embryos (Figure 3M and 3N). This demonstrates that Pbx proteins may function either directly or indirectly as a key repressor of *otx2 *expression. Other genes with known retinal gene expression were assayed but did not show Pbx dependence, including *efnB2a *and *ephA4b *(data not shown). Taken together, these data demonstrate a clear role for Pbx proteins in regulating gene expression within the vertebrate retina, especially within the dorsal region.

### The role of Meis proteins in regulating retinal gene expression

**Figure 4:**
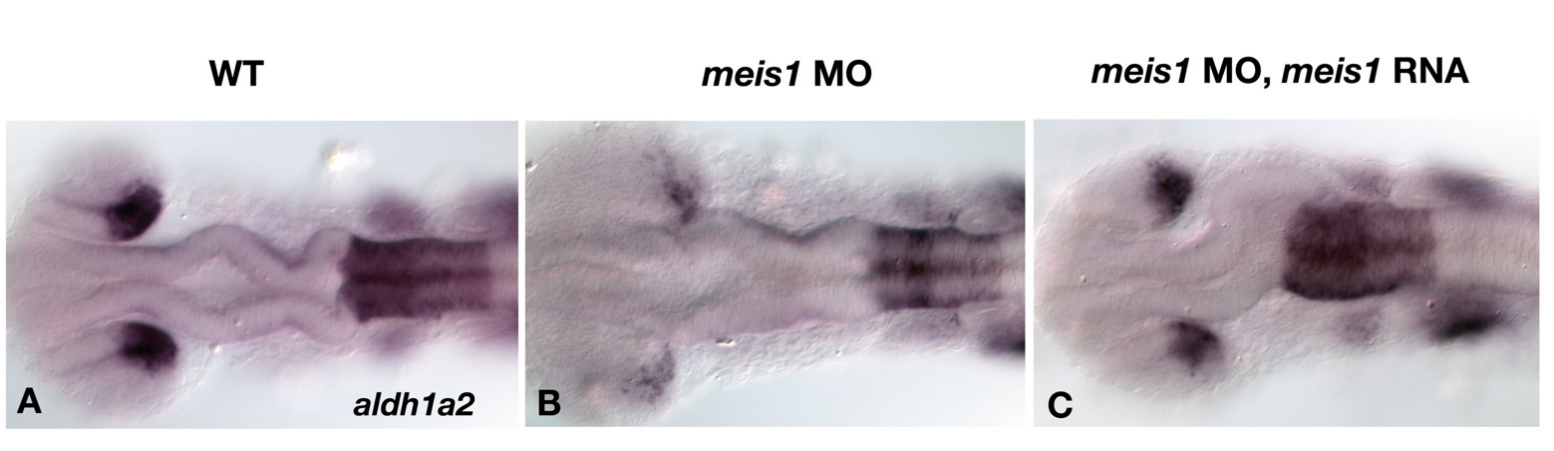
*meis1.1 *is required for expression of *aldh1a2*. The expression of *aldh1a2 *is reduced in *meis1.1 *morphants (B) when compared to wild type (A) at 24 hpf. As a control for morpholinos specificity, injection of *meis 1.1 *mRNA rescues this phenotype (compare B and C). All embryos were also hybridized with *hoxa2b *as well as *aldh1a2*.

Within the hindbrain, Pbx cooperates with Hox and Meis/Pknox proteins to drive expression of target genes. Although *hox *genes are not expressed in the retina, *meis/pknox *genes are expressed strongly in this tissue [[1,10-12] and unpublished data]. Furthemore, *pknox1 *hypomorphs and *Meis1 *knockout mice have morphological defects in retinal formation [16,18]. As *meis1.1 *is specifically expressed in the retina during early eye patterning in zebrafish [1,25], we utilized a morpholino to knockdown Meis1.1 function. This treatment results in the reduction of *aldh1a2 *expression in the retina and *hoxa2b *expression in the hindbrain at 24 hpf (Figure 4B). Co-injection of MO-insensitive *meis1.1 *mRNA is sufficient to restore *aldh1a2 *and *hoxa2b *expression in *meis1.1 *morphants (Figure 4C), indicating the specificity of the morpholino phenotype. The reduction of retinal *aldh1a2 *expression in *meis1.1 *morphants is very similar to *pbx2/4 *null embryos and is thus consistent with the idea that Meis and Pbx proteins cooperate to regulate transcription of this particular gene.

### Pbx is required for both early and late eye patterning genes

**Figure 5:**
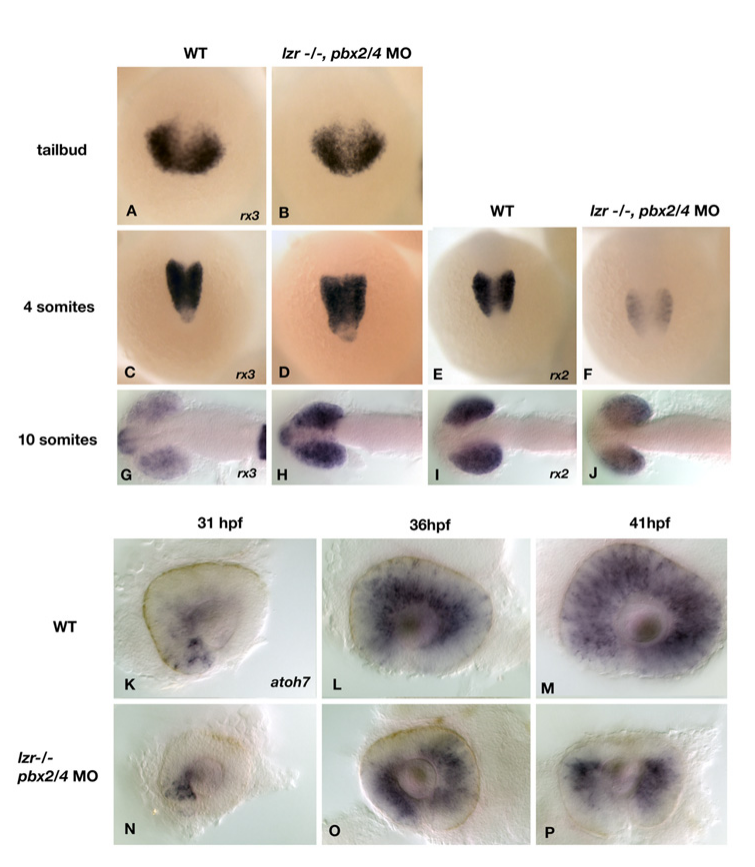
The expression of *rx2*, *rx3*, and *atoh7 *is aberrant in *pbx2/4 *null embryos. The expression of *rx3*, the earliest known eye field marker is unaffected at the tailbud and 4 somite stage (A-D) but is increased in expression at the 10 somite stage (H) when compared to wildtype (G). The expression of *rx2 *is reduced at both the 4 and 10 somite stage in *pbx2/4 *null embryos (F and J), when compared to wildtype (E and I). No expression of *rx2 *is observed at the tailbud stage. The expression of *atoh7 *was analyzed as a marker of differentiating retinal ganglion cells. In wild type embryos, expression begins in the ventral nasal domain of the retina at about 31 hpf (K). Expression proceeds in a wave-like fashion to include the dorsal retina by 36 hpf (L), and has filled the entire retina by 41 hpf (M). In *pbx2/4 *null embryos, expression is initiated correctly at 31 hpf (N). Expression proceeds in a wave-like fashion but is excluded from the dorsal domain of the retina at both 36 hpf (O) and 41 hpf (P).

To determine when Pbx proteins are first required for eye patterning, we analyzed *retinal homeobox (rx) *gene expression in *pbx2/4 *null embryos at early stages of development. The expression of *rx *genes are limited to the eye fields during early development, and expression is required for proper eye development in zebrafish as over-expression leads to ectopic eye formation [26]. *rx3 *is expressed in the optic primordium at the tailbud stage (Figure 5A), while both *rx2 *and *rx3 *are expressed after somitogenesis (Figure 5C, G, E, and 5I). In *pbx2/4 *null embryos, the expression of *rx3 *appears unaffected during early eye specification, but has failed to be downregualted by 10 somites (21/28 embryos, Figure 5H). The expression of *rx2*, however, is reduced throughout somitogenesis in *pbx2/4 *null embryos (17/20 embryos at 4 somites, 18/23 at 10 somites, Figure 5F and 5J). Thus, *pbx *gene function plays a key role in the specification of early eye tissue.

It is likely that *pbx2/4 *function is also needed later in eye development as processes such as retinal ganglion cell outgrowth are also affected when Pbx function is inhibited. We therefore analyzed the expression of *atonal homolog 7 (atoh7)*, which is expressed in a wave-like fashion (Figure 5K–M) in the inner retina prior to retinal ganglion cell differentiation [27]. There is a marked reduction in the expression of *atoh7 *in the dorsal retina at both 36 hpf (18/27 embryos) and 41 hpf (22/32 embryos) (Figure 5O and 5P), while expression in the rest of the inner retinal tissue remains unaffected. This indicates that a lack of *atoh7 *in the dorsal retina may affect retinal ganglion cell differentiation in this domain, which may contribute to the observed defects in retinal ganglion cell outgrowth.

### Pbx proteins regulate polarized gene expression in the optic tectum

**Figure 6:**
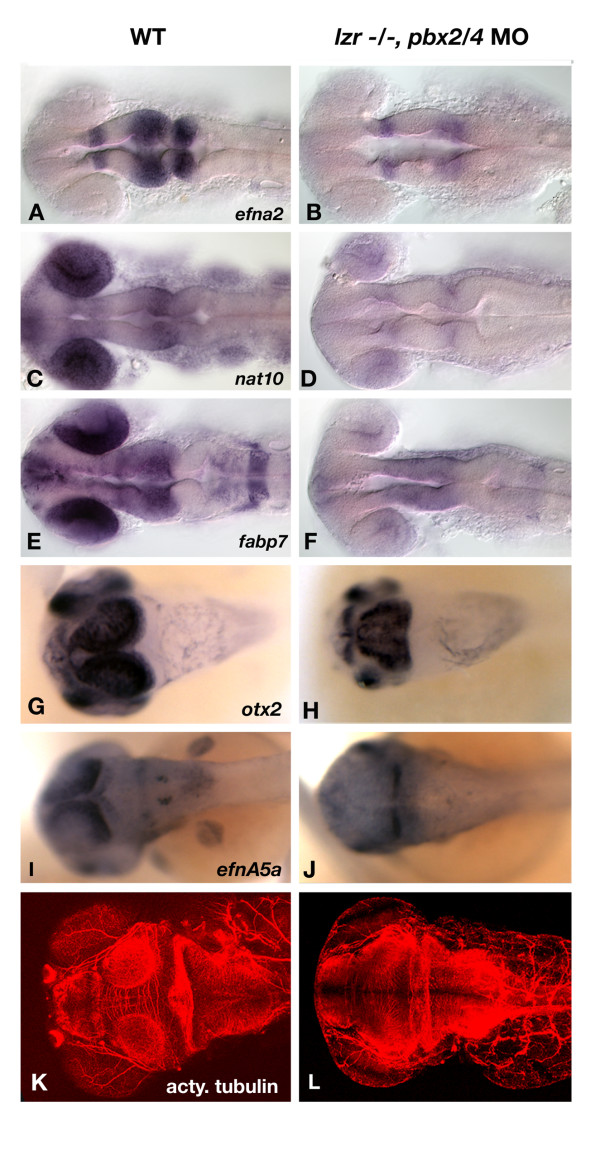
Aberrant tectal patterning is observed in *pbx2/4 *null embryos. *efna2 *expression includes the posterior forebrain, the tectum, and the anterior hindbrain in wild type embryos (A), and is reduced in *pbx2/4 *null embryos (B) at 24 hpf. The expression of both *nat10 *and *fabp7a *includes both the eye and tectum in wild type embryos (C) and (E), and is almost completely absent in both regions in *pbx2/4 *null embryos (D and F). *otx2 *is expressed in the optic tectum at 48 hpf, specifically toward the periphery of the tectum with lower levels observed immediately adjacent to the eye (G). In *pbx2/4 *null embryos, there is a distinct change in expression domain in which the highest levels of expression are observed immediately adjacent to the eye (H). The gradient of *efnA5a *expression, which decreases toward the anterior (I), is attenuated at 48 hpf in *pbx2/4 *null embryos (J). The acetylated tubulin antibody marks the axons of the optic tectum at 3 dpf (K), which is smaller and disorganized in *pbx2/4 *null embryos (L).

Previous research has demonstrated a role for Pbx in patterning both the midbrain-hindbrain and diencephalic-mesencephalic boundaries. Consistent with this role, our microarray identified three putative Pbx dependent transcripts are expressed within the tectum. These genes, *ephrin A2 *(*efna2*), *fatty acid binding protein 7a *(*fabp7a*), *N-acetyltransferase 10 *(*nat10*) were examined by whole mount *in situ *hybridization to confirm the results of the microarray experiment. *efna2 *is expressed in the diencephalon and tectum (Figure 6A). The other two genes, *fabp7a *and *nat10*, have significant expression in the retina as well as the tectum (Figure 6C and 6E). Each transcript is downregulated in *pbx2/4 *null embryos with residual expression of each gene found at the midbrain-hindbrain boundary (Figure 6B, D, F). This is consistent with previous findings showing that Pbx proteins are required for the maintenance of gene expression in the mesencephalon.

As development proceeds, the tectum becomes highly organized and is characterized by the polarization of gene expression. At 48 hpf, the expression of *otx2 *and *ephrinA5a (efnA5a) *is strongest at the periphery of the tectum, with the highest expression observed distal to the eye while decreasing proximally (Figure 6G and 6I). These gradients are disrupted in *pbx2/4 *null embryos, whereby expression of *otx2 *is greatest proximal to the eye (Figure 6H), and expression of *efnnA5a *is confined to the posterior tectum (Figure 6J). By 3 dpf, the optic tectum is smaller and disorganized in *lzr-/-*, *pbx2/4 *morphant embryos (Figure 6L), as shown by visualization of acetylated tubulin-positive axons. These data clearly indicate a requirement for Pbx proteins in patterning the optic tectum.

### Pbx is critically required for expression of *gdf6a*, itself a regulator of dorsal retina patterning

**Figure 7:**
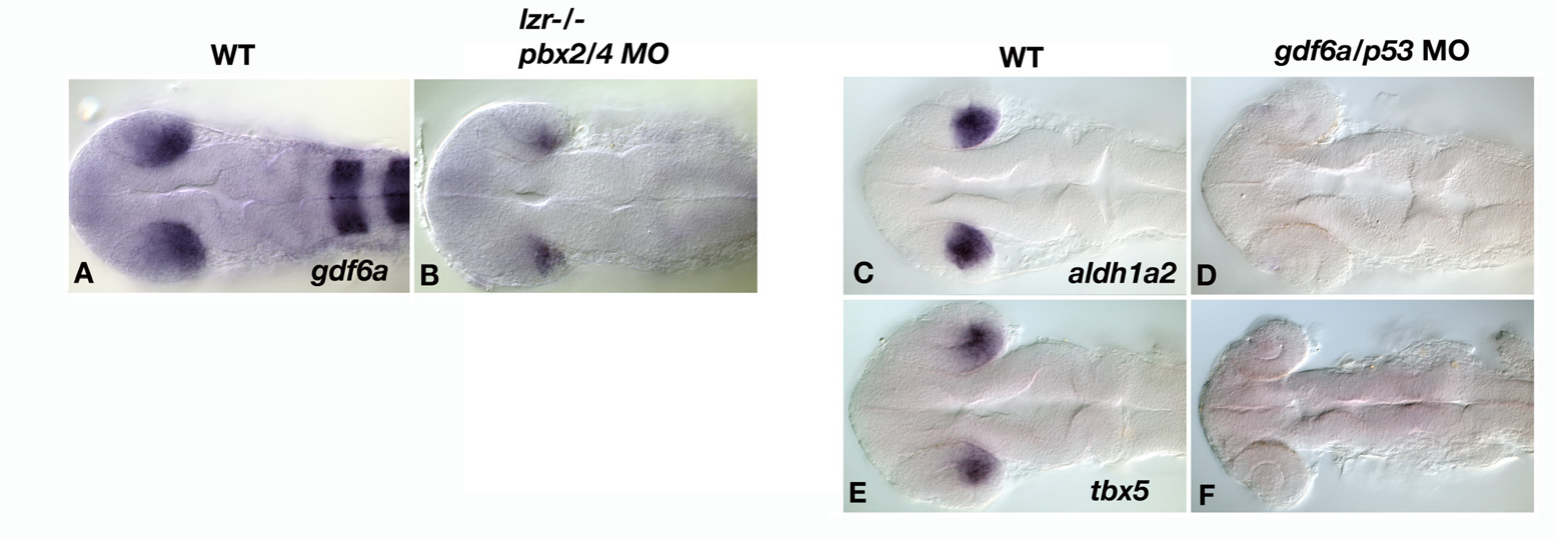
*gdf6a *is genetically downstream of *pbx2/4 *and is necessary for expression of genes in the dorsal/temporal retina. Expression of *gdf6a *is strongly reduced in *pbx2/4 *null embryos (B), when compared with wildtype (A) at 24 hpf. Knockdown of *gdf6a *results in a loss of *aldh1a2 *and *tbx5 *expression (D and F) when compared with wildtype (C and E). *pbx2/4 *null embryos were also hybridized with *egr2b *as well as *gdf6a*, as a marker of *pbx *function.

Gdf6a is a growth factor of the BMP family, whose retinal expression is restricted to the temporal/dorsal domain. Knockdown of *gdf6a *results in smaller eyes, as well as reduced expression of eye markers in the dorsal/temporal retina [28]. As such, we analyzed whether *pbx *and *gdf6a *function in the same pathway to regulate eye patterning, and further analyzed the expression of eye markers in *gdf6a *morphants. A knockdown of *gdf6a *results in the elimination of *aldh1a2 *and *tbx5 *expression (Figure 7D and 7F), markers of the dorsal/temporal retina. As the expression of genes in the dorsal/temporal retina is similar in *gdf6a *and *pbx2/4 *null embryos, we tested whether *gdf6a *functions in the same genetic pathway as *pbx*. When Pbx2/4 function is eliminated, the expression of *gdf6a *is strongly reduced (Figure 7B), indicating that *gdf6a *functions genetically downstream of *pbx*. The expression of *pbx4 *is not affected in *gdf6a *morphants (data not shown), further supporting the idea that *gdf6a *functions genetically downstream of *pbx*.

## Discussion

In this paper, we present evidence that *pbx2/4 *gene function is essential for proper retinal ganglion cell axon pathfinding. This phenotype likely results from aberrant Pbx mediated gene transcription in both the eye and tectum of *pbx2/4 *null embryos. Although the eye laminar structure is relatively normal after 5 days of development, connections between the RGC axons and the optic tectum are not made. Through microarray and *in situ *analysis, we have identified numerous candidate genes that may contribute to RGC axon guidance phenotype seen in embryos with reduced Pbx function. Abnormalities in eye patterning begin during early somitogenesis, and persist throughout development. Defects in the differentiation of retinal ganglion cells may also contribute to this phenotype, as the expression of *atoh7 *is eliminated in the dorsal region of the neural retina in *pbx2/4 *null embryos. The data presented here builds on current knowledge of Pbx proteins in embryonic patterning, and provides a novel role for Pbx in patterning the eye and tectum, and subsequently, the regulation of RGC axon outgrowth and pathfinding.

An absence of Pbx function results in reduced expression of genes involved in eye patterning. In *pbx2/4 *null embryos, defects are seen in the expression of *rx2 *during early somitogenesis, and at 10 somites, *rx3 *expression has not been downregulated as seen in wild type embryos [26]. This could lead to defects in retinal ganglion cell and photoreceptor development, as *rx2 *and *rx3 *are also expressed in these subsets of cells later in development. We have identified *aldh1a2*, *hmx4*, and *tbx5 *as Pbx-dependent transcripts at 18 hpf. Each of these genes has the potential to play a strong role in regulating retinal patterning. For example, *aldh1a2*, which is involved in the synthesis of retinoic acid, is likely to affect development of the visual system in zebrafish as retinoic acid is required for development of photoreceptor cells and pigmented epithelium in the developing zebrafish eye [29]. By 3 dpf, the photoreceptor layer is highly disorganized in fish with reduced *pbx *gene function, indicating a possible effect of *aldh1a2 *misexpression. Moreover, we also observed a downregulation of *tbx5 *via both microarray and *in situ *analysis, a downstream effector of retinoic acid signaling that is essential for establishment of the dorsoventral boundary of the eye [30,31].

It has been documented that the homeodomain transcription factor *soho1 *represses the expression of *epha3 *in chicks, and is necessary for eye development. The zebrafish Pbx dependent gene *hmx4 *is likely the functional ortholog of chick *soho1*, since it is expressed in the retina and shares extensive sequence similarity. misexpression of *soho1 *in chicks affects *epha3 *expression [32], and loss of *epha3 *leads to premature arborization by RGC axons that originate in the nasal retina, while those that originate in the posterior retina appear unaffected [33]. In *pbx2/4 *null embryos, *hmx4 *is down regulated during early eye patterning, and thus may also play a role in phenotypes observed in eyes with reduced *pbx *gene function, such as a lack of RGC axon outgrowth.

Given the profound changes in gene expression, it is surprising that laminar structure and positioning of the optic nerve is relatively normal without Pbx2/4 function. As such, there is no evidence for overt dorsal/ventral patterning defects or improper cell differentiation. We believe a partial answer to this paradox lies in the data from the 28 hpf microarray, in which each of these Pbx-dependent transcripts has more normal levels of expression (data not shown). We confirmed these results with *in situ *analysis, showing that changes in *aldh1a2 *and *tbx5 *expression are more subtle, while the expression of *hmx4 *is near wild-type levels at 24 hpf, indicating that Pbx2/4 are essential for proper initiation of these transcripts, but that other factors are capable of regulating their transcription in the absence of Pbx2/4. At 24 hpf, other *pbx *genes, including *pbx1 *and *pbx3.1*, are expressed in the zebrafish embryo. As such, these Pbx proteins or other transcription factors might also regulate eye patterning genes at 24 hpf.

Although not identified in our microarray screen for Pbx dependent transcripts, we analyzed the expression of *otx2*, which in wild type embryos, is expressed throughout the optic vessicle early in development [23], but is restricted to the RPE between 22–24 hpf. *otx2 *is also strongly expressed in the optic tectum, and could therefore be responsible for axon guidance through either eye or tectal patterning. We noticed an expansion of expression to include the entire eye in *pbx2/4 *null embryos at 24 hpf, indicating that these embryos are unable to restrict the expression of *otx2 *as development proceeds. A loss of retinoid signaling in mice leads to reduced expression of *tbx5 *and expanded expression *otx2 *throughout the retina, placing both genes downstream of *aldh1a2 *[34]. This provides a possible signaling mechanism for the expansion of *otx2 *in *pbx2/4 *null zebrafish whereby Pbx is required for expression of *aldh1a2*, which, in turn, is required for the restriction of *otx2 *to the RPE. As restriction of *otx2 *expression is required for specification of the RPE layer, it is logical that *pbx2/4 *null embryos would have defects in the specification of RPE cells. In both *lzr *and *pbx2/4 *null embryos, the RPE is thickened in a subtle, but reproducible manner. Thus it is likely that the ectopic expression of *otx2 *in *pbx2/4 *null embryos leads to the thickening of the RPE, but aberrant expression of other retinal transcripts may also be involved.

Many Pbx functions, such as hindbrain patterning and blood development, are known to involve Meinox binding partners. Meinox proteins can stabilize Pbx proteins, and are often essential for Pbx function through cooperative DNA binding. [1,9]. *Meis1 *knockout mice and *Prep/Pknox *mutants have morphological defects in retinal formation including an inappropriately positioned optic nerve [16]. The position of the optic nerve implies that *Meis1 *is required for proper dorsal-ventral patterning of the retina, although marker analysis has yet been performed. To create zebrafish with reduced Meis1.1 function, we injected embryos with *meis1*.*1 *translation blocking morpholinos. As with *pbx2/4 *null embryos, knockdown of Meis1.1 function reduced or eliminated *aldh1a2 *expression. Thus, it is likely that Pbx2/4 and Meis1.1 act cooperatively to regulate at least some aspects of eye patterning. This is in concert with previously published reports that indicate that both proteins are needed to pattern tissues such as the hindbrain [1,9].

Using our microarray approach, we have also identified reduced tectal expression of *fabp7a*, *nat10*, and *efna2 *in *pbx2/4 *null embryos. Interestingly, *fabp7a *and *nat10 *also show decreased expression in the eye in *pbx2/4 *null embryos, and thus may be important in patterning both structures in a Pbx dependent fashion. *FABP7 *has been identified as a downstream target of Pbx/Meinox signaling in humans, whereby increased expression of PKNOX1 (human *prep1 *homologue) leads to overexpression of *FABP7 *in fetal trisomy leading to Down's Syndrome [35].

Further in development, the optic tectum becomes highly polarized in wild type embryos, which is required for proper retinal ganglion cell axon outgrowth and mapping. By 48 hpf, tectal gradients of *otx2 *and *ephrinA5a *expression have been disrupted in embryos with reduced Pbx function, indicating its vital role for patterning this tissue. Given these results, it is possible that RGC axon pathfinding defects may result in part from *pbx *dependent patterning defects in the tectum in concert with *pbx *patterning defects in the retina. An alternative hypothesis would indicate that forebrain patterning, specifically via *sonic hedgehog (shh) *signaling could lead to the observed axon outgrowth defect, as *shh *has been shown to negatively regulate growth cone movement [36]. This is not the case in *pbx2/4 *null embryos as no changes in *shh *expression, nor two of its downstream target genes *titf1a *and *titf1b*, are observed (data not shown).

It has recently been reported that a knockdown of *gdf6a*, a growth factor of the BMP family, results in smaller eyes in both zebrafish and frogs [28,37]. Furthermore, the expression of marker genes in the dorsal/temporal domain of the retina is dependent on Gdf6a function. We have expanded on the marker analysis in *gdf6a *morphants to include *aldh1a2 *and *tbx5*, which are both eliminated in *gdf6a *morphants. Moreover, *gdf6a *expression is strongly reduced in *pbx2/4 *null embryos, indicating that *gdf6a *acts genetically downstream of both *pbx*, and is essential for patterning the dorsal/temporal retina. As similar phenotypes are seen with ectopic expression of FGF3/8 in the temporal retina [38], it is likely that FGFs and GDFs represent opposing signals to pattern the nasal and temporal domains of the retina, respectively. Given that our data suggest that Pbx proteins are genetically upstream of *gdf6a *expression in the retina, it appears that Pbx may play an important role in regulating the Fgf-Gdf antagonism that leads to correct retinal patterning.

## Conclusion

The results presented support a model where aberrant eye and tectal patterning in *pbx2/4 *null embryos leads to RGC axon outgrowth errors. Eye patterning in the dorsal-temporal domain of the retina involve the function of *meis1.1 *and a downstream growth factor, *gdf6a*.

## Methods

### Zebrafish strains, morpholinos, and *in situ *hybridization

The mutant *pbx4 *allele (b557), also known as *lazarus, (lzr)*, was identified by altered expression patterns of *egr2b (krox20) *in the developing hindbrain, and has been described previously [8]. For the microarray experiments, *pbx2/4 *null embryos were created by injecting *pbx2 *(CCGTTGCCTGTGATGGGCTGCTGCG) translation blocking morpholinos into one-cell maternally and zygotically mutant *lzr *embryos (mz *lzr*). Germ line transplantation was used to create mz *lzr *embryos and has been described previously [2]. For all other experiments, *pbx2/4 *null embryos were created by injecting *pbx2 *and *pbx4 *(AATACTTTTGAGCCGAATCTCTCCG) translation blocking morpholinos into one-cell zygotically mutant *lzr *embryos. These embryos are phenotypically indistinguishable from mz *lzr *embryos injected with *pbx2 *morpholinos. Approximately 3 nl of *pbx2/4 *morpholino was injected at a concentration of 1 mg/ml. To create a similarly mispatterned embryo for microarray studies, we injected *lzr *embryos with morpholinos directed against *hoxb1a *(GGAACTGTCCATACGCAATTAA) and *hoxb1b *(AATTCATTGTTGACTGACCAAGCAA and ACCAAGCAAAATTGATTAAGCAGGG).

For *gdf6a *knockdown, a splice blocking morpholino (GCAATACAAACCTTTTCCCTTGTCC) was utilized. This morpholino results in the production of *gdf6a *messenger RNA containing its lone intron. This allows for normal maternal *gdf6a *function, as *gdf6a *is required for patterning the early embryo, but inhibits zygotic *gdf6a *function prior to eye development. As high levels of necrosis are observed in *gdf6a *morphants, *gdf6a *morpholinos were co-injected with a *p53 *translation blocking morpholino (GCGCCATTGCTTTGCAAGAATTG) [39]. To block *meis1.1 *function, we injected a translation-blocking morpholinos (GTATATCTTCGTACCTCTGCGCCAT) into 1-cell zebrafish embryos. To rescue the *meis1.1*MO phenotype, we injected approximately 200 pg of 6X-myc tagged *meis1.1 *mRNA synthesized in vitro using the Ambion SP6 mMessage machine kit.

Whole mount *in situ *hybridization analysis was performed essentially as previously described [40], with the following modifications: probes were not hydrolyzed; Proteinase K treatment (10 ug/ml) was performed for 30 seconds (tailbud- 10 somite embryos), 3 minutes (18–24 hpf embryos), and 20 minutes (embryos between 24–48 hpf). Embryos were manually deyolked, and photographed using a Zeiss AxioImager Z1 compound microscope and an Axiocam HR digital camera or on an Olympus steroscope with a Qimaging micropublisher camera. Embryos were raised at 25–31°C and staged according to published staging hallmarks [41].

### cDNA microarray analysis

For each microarray hybridization, target cDNA was synthesized from either mz *lzr*, or *lzr *embryos injected with either *pbx2 *or *hox-1 *morpholinos, respectively. cDNA was generated from RNA templates isolated at both 18 hpf and 28 hpf. cDNA was labeled with either Cy3 or Cy5, and hybridized to microarrays containing 8448 cDNA probes, including 768 controls spots (e.g. negative, positive, and normalization controls). An Axon scanner (Molecular Devices) and Genepix software (Molecular Devices) were used to determine the intensities of each spot and normalize the data. All experiments were conducted in biological duplicate while simultaneously switching fluorescent labeling compounds.

### Identification of *hmx4*

A previously uncharacterized EST clone was identified by microarray analysis as having reduced expression in *pbx2/4 *null embryos. *in situ *analysis identified expression in the lens and dorsal retina. The clone was sequenced and genomic location identified in zebrafish, and compared to sequences in other organisms. The EST (DY55561.1), is highly similar to that of *hmx3 *in zebrafish (55% amino acid identity), and is also highly similar to chick *soho1 *(59% amino acid identity). Based on the homology of the homeodomain and genomic organization, we have named this gene *hmx4*.

### Eye cross sections

Wild type and *pbx2/4 *morpholino injected embryos were grown to 5 dpf, then fixed in 4% PFA. Embryos were embedded in Spurr's Resin for 1 μm ultramicrotome sectioning with a diamond blade, and stained with Richardson's blue for visualization of retinal layers. Sections were visualized using Zeiss Axioimager Z1 coumpound microscope and photographed with Zeiss Axiocam HR. Eye retinal pigmented epithelium and total eye area were quantified using ImageJ software.

### DiI and DiO RGC labeling

Two lipophylic dyes, DiI and DiO (Molecular Probes), were used to label retinal ganglion cell axons. DiI was dissolved in dimethylformamide (DMF), at a concentration of 12.5 mg/ml. DiO was dissolved in chloroform at a concentration of 12.5 mg/ml. Embryos were raised in 0.0015% phenylthiourea (PTU) to inhibit pigment formation and fixed in 4% paraformaldhyde at 3.5 and 5.5 days. Embryos were mounted dorsal side up in 1% in low melting point agrose for injections. DiI was injected into the nasal domain of the right eye, while DiO was injected into the temporal domain of the right eye. Embryos were removed form the agrose and incubated in PBS 0.01% tween20 overnight at 28°C. Embryos were flat mounted in 70% glycerol, dorsal side up. Imaging was accomplished with a SP2 confocal microscope (Leica) using 20X objective.

### Immunohistochemistry

The acetylated tubulin antibody (Sigma-Aldrich) staining protocol has been published previously [42]. Briefly, zebrafish embryos (3 dpf) were fixed in Dent's fix (80% methanol/20%DMSO), and primary antibody incubation was completed overnight at 4°C (1:500) after blocking in PBS-DBT (1% DMSO/1% BSA/0.5% tween-20, 10% goat serum). After 5 washes in PBS 0.5% tween, a secondary antibody (anti-mouse Alexaflour 546, Molecular Probes, 1:1000), was added and incubated overnight at 4°C.

For zpr-1 (1:200) staining, embryos were fixed in 4% PFA overnight at 4°C. Embryos were blocked in PBS 2% goat serum, 2 mg/ml BSA, 0.1% triton X-100. After washing in PBSDTT, a secondary antibody (anti-mouse Alexaflour 488, Molecular Probes) was diluted 1:1000 and incubated overnight at 4°C. Embryos were mounted in 70% glycerol for imaging on a SP2 confocal microscope (Leica) using both 20X and 40X (oil) objective.

## Authors' contributions

CRF was responsible for RGC labeling assays and *in situ *analysis in *pbx2/4 *null embryos, *gdf6 *morphants, immunohistochemistry, and writing of the manuscript. TE was responsible for *meis1.1 *morphant analysis, and editing the manuscript. DC and RK were responsible for eye cross sections in *pbx2/4 *null embryos. KB was involved in *in situ *analysis in *pbx2/4 *null embryos and was involved in the microarray studies. DH, JS, KWS, JN, CBM, and AJW were involved in the microarray analysis and subsequent *in situ *hybridization verifications. AJW is the principal investigator.
